# Application of machine learning combined with population pharmacokinetics to improve individual prediction of vancomycin clearance in simulated adult patients

**DOI:** 10.3389/fphar.2024.1352113

**Published:** 2024-03-18

**Authors:** Guodong Li, Yubo Sun, Liping Zhu

**Affiliations:** ^1^ Department of Mathematics, Guilin University of Electronic Technology, Guilin, China; ^2^ Department of Mathematics, Changji University, Xinjiang, China

**Keywords:** vancomycin, population pharmacokinetics, machine learning, clearance, prediction

## Abstract

**Background and aim:**

Vancomycin, a glycopeptide antimicrobial drug. PPK has problems such as difficulty in accurately reflecting inter-individual differences, and the PPK model may not be accurate enough to predict individual pharmacokinetic parameters. Therefore, the aim of this study is to investigate whether the application of machine learning combined with the PPK method can improve the prediction of vancomycin CL in adult Chinese patients.

**Methods:**

In the first step, a vancomycin CL prediction model for Chinese adult patients is given by PPK and Hamilton Monte Carlo sampling is used to obtain the reference CL of 1,000 patients; the second step is to obtain the final prediction model by machine learning using an appropriate model for the predictive factor and the reference CL; and the third step is to randomly select, in the simulated data, a total of 250 patients for prediction effect evaluation.

**Results:**

XGBoost model is selected as final machine learning model. More than four-fifths of the subjects’ predictive values regarding vancomycin CL are improved by machine learning combined with PPK. Machine learning combined with PPK models is more stable in performance than the PPK method alone for predicting models.

**Conclusion:**

The first combination of PPK and machine learning for predictive modeling of vancomycin clearance in adult patients. It provides a reference for clinical pharmacists or clinicians to optimize the initial dosage given to ensure the effectiveness and safety of drug therapy for each patient.

## 1 Introduction

Vancomycin, a glycopeptide antimicrobial drug, has a good therapeutic effect on Gram-positive bacteria such as methicillin-resistant *Staphylococcus aureus* (MRSA). It is characterized by a narrow therapeutic window and large individual differences, so it needs to be administered individually, and blood concentration monitoring (TDM) is often required in the clinic to improve the therapeutic efficacy and the incidence of adverse reactions (Ye et al., 2016). However, the selection of vancomycin pharmacokinetic parameters remains controversial, including trough concentration, clearance, etc (Ghasemiyeh et al., 2023).

Population pharmacokinetics (PPK), which combines classical pharmacokinetic modeling with population statistical modeling. Vancomycin has been the subject of a number of PPK studies in adults (Aljutayli et al., 2020; Lindley et al., 2023). It has been shown that population pharmacokinetic modeling of vancomycin in Chinese adult patients can be established (He et al., 2014; Gao et al., 2018). PPK study of vancomycin is important for guiding clinical dosing. However, PPK model may not be accurate enough to predict individual pharmacokinetic parameters.

Machine learning (ML) is a data-driven approach that uses training data to learn how to accomplish tasks through various algorithms and then make decisions and predictions about specific events. In pharmacokinetics, machine learning allows for analysis and prediction (Ota and Yamashita, 2022; Wang et al., 2023). The combination of machine learning and population pharmacokinetics is a new tool for drug research and development (Zhu et al., 2022; Damnjanovic et al., 2023). It has been reported that machine learning combined with PPK method can improve the prediction of individual clearance of six drugs in neonates (Tang et al., 2021). However, there are few similar studies in adult patients.

Nowadays, in the field of machine learning, many models are produced. Decision Tree Regression Model (Kaminski et al., 2018), is a regression algorithm that uses decision tree as the basic model, which fits the data by dividing the input variables into multiple features and constructing a decision tree based on these features. Gradient Boosting Decision Tree (Si et al., 2017), is an algorithm based on integration learning, which fits the data by integrating multiple decision tree models together. Extreme Gradient Boosting (Chen and Guestrin, 2016), is an efficient gradient boosting algorithm which minimizes the loss function by using a greedy algorithm to select the optimal features for splitting. Extra Tree Regression Model (Geurts et al., 2006), is an integrated learning algorithm that mitigates the variance of the model by averaging the predictions of multiple decision trees to improve the robustness of the model.

The aim of this study is to investigate whether the application of machine learning combined with the PPK method could improve the prediction of vancomycin CL in Chinese adult patients and provide a reference for the individualized dosing of vancomycin in Chinese adult patients.

## 2 Methods

### 2.1 Flow of the study

**FIGURE 1:**
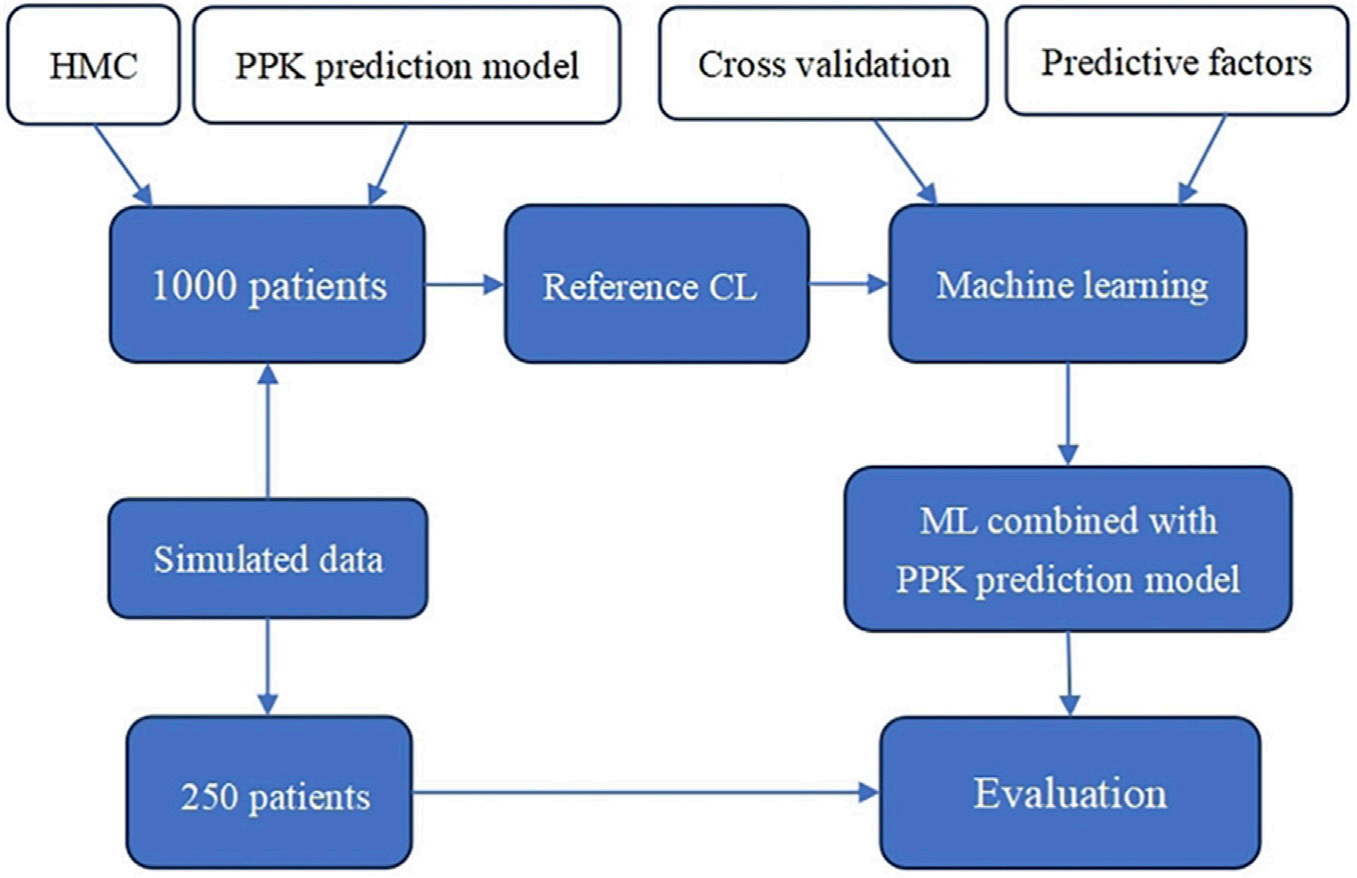
Flow chart of the study.

The flow chart of this study is shown in Figure 1. First, the reference CL of 1,000 patients is obtained using PPK and Hamilton Monte Carlo (HMC) sampling. Then, machine learning is performed on the predictors (basic patient information) and the reference CL using an appropriate model to obtain the ML combined with PPK prediction model. Finally, in the simulated data, 250 patients are randomly selected for prediction effect assessment.

### 2.2 Patients data

The simulated data are provided by Guangzhou Jingyuan Pharmaceutical Company and contained sex, age, weight, serum creatinine (Scr) and simulated vancomycin CL.

The number of patients is set at 250 in the machine learning test group and the evaluation. Also, since the ratio of the training group to the test group in machine learning is 1:3, the number of patients participating in machine learning is set at 1,000, which are randomly selected from the simulated data. The patients participating in the evaluation are also randomly selected from the simulated data.

### 2.3 Population pharmacokinetics

This study is conducted to explore whether machine learning combined with the PPK method could improve the prediction of vancomycin CL in adult patients. For the PPK model, the vancomycin PPK model for adult patients from the literature (He et al., 2014) is used, in which the CL prediction equation is used to calculate the vancomycin CL for each simulated patient. The values of each variable in the simulated data satisfy the requirements of the model. The CL prediction equation is shown in Eq. 1. The vancomycin CL calculated for each patient by this equation was defined as the reference CL for each patient.

$$CL=\left[1.71\times e^{\eta_{1}}+8.31\times \left(1-e^{-0.0113\times e^{\eta_{2}}\times CLcr}\right)\right]\times 0.475^{\frac{Age}{72}}$$ (1)

$$\eta_{1}∼N\left(0,0.415^{2}\right),\eta_{2}∼N\left(0,0.381^{2}\right)$$

(Where CLcr denotes creatinine clearance, $\eta_{1}$ and $\eta_{2}$ denote Variability between patients).

### 2.4 Machine learning

To fit the obtained reference CL, four machine learning models are selected for exploratory analysis based on rules of thumb and literature review, namely, Decision Tree Regression (DTR), Gradient Boosted Decision Tree (GBDT), eXtreme Gradient Boosting (XGBoost), and Extra Tree Regression (ETR). Each machine learning model is implemented through computer simulation.

In machine learning, the input predictive factors are age, weight, serum creatinine, and gender. To get reliable and stable models, Cross-validation is done for all the four machine learning models. Based on cross-validation, the dataset of 1,000 simulated patients are randomly divided into a test set and a training set in a ratio of 1:3. That is, in the machine learning of this paper, the training set contains 750 patients and the test set contains 250 patients.

### 2.5 Selection of machine learning models

Four statistical metrics are chosen to select the most suitable machine learning model for prediction: the coefficient of determination ( $R^{2}$ score), the mean square error (MSE), the root mean square error (RMSE), and the mean absolute error (MAE).

The coefficient of determination ( $R^{2}$ score), which reflects the proportion of the total variation in the dependent variable that can be explained by the independent variable through the regression relationship, is a kind of evaluation index for the assessment of linear models. The maximum value is one and the minimum value is 0. When the value of the coefficient of determination is closer to 1, it means that the model fits better; when the value is closer to 0, it means . The equations for calculating the coefficient of determination are shown in Eqs 2–5.

$$R^{2}=1-\frac{SSE}{SST}$$ (2)

$$SSR=\sum_{i=1}^{n}{\left({\hat{y}}_{i}-\bar{y}\right)}^{2}$$ (3)

$$SSE=\sum_{i=1}^{n}{\left(y_{i}-{\hat{y}}_{i}\right)}^{2}$$ (4)

$$SST=SSR+SSE=\sum_{i=1}^{n}{\left(y_{i}-\bar{y}\right)}^{2}$$ (5)

(Where SSR denotes the sum of squared regressions, SSE denotes the sum of squared residuals, SST denotes the sum of squared total deviations. $y_{i}$ denotes the reference clearance for the *i*th patient, $\bar{y}$ denotes the mean of the reference clearance for 1,000 patients, and ${\hat{y}}_{i}$ denotes the predicted clearance for the *i*th patient).

The mean square error (MSE), which is the average of the deviations between the theoretical and the actual observed values, is a measure of the degree of difference between the estimated and the estimated quantity. It is the most general criterion for evaluating point estimates. Root Mean Square Error (RMSE), the square root of the mean square error, is used for the same computational purpose, but with more emphasis on the magnitude of the error. The Mean Absolute Error (MAE), which is the average of the absolute values of the deviations of all individual observations from the arithmetic mean, avoids the problem of canceling out errors and thus accurately reflects the magnitude of the actual prediction error. The smaller the value of mean square error, root mean square error and average absolute error, the better the prediction effect of the model. The larger the value, the worse the prediction effect of the model. The equations for the mean square error, root mean square error, and mean absolute error are shown in Eqs 6–8.

$$MSE=\frac{1}{n}\sum_{i=1}^{n}{\left({\hat{y}}_{i}-y_{i}\right)}^{2}$$ (6)

$$RMSE=\sqrt{\frac{1}{n}\sum_{i=1}^{n}{\left({\hat{y}}_{i}-y_{i}\right)}^{2}}$$ (7)

$$MAE=\frac{1}{n}\sum_{i=1}^{n}\left|{\hat{y}}_{i}-y_{i}\right|$$ (8)

### 2.6 Evaluation

To evaluate the predictive performance of the final model, 250 patients are randomly selected from the simulated data. The combination prediction model and the original PPK model are used to predict the clearance of vancomycin in these patients, respectively. Two types of prediction results are obtained and compared with the simulated actual clearance rate for evaluation. The absolute and relative errors between the actual and predicted clearance rates are calculated for these patients.

Absolute errors, refers to the absolute value of the difference between the true value and the measured value, can indicate the reliability of a measurement result. Relative error, is the value obtained by the ratio of absolute error to true value, and it can compare the reliability of different measurement results. The equations for absolute and relative errors are shown in Eqs 9, 10.

$$Absolute\;errors=\left|CL_{prediction}-CL_{true}\right|$$ (9)

$$Relative\;errors=\frac{\left|CL_{prediction}-CL_{true}\right|}{CL_{true}}$$ (10)

(Where $CL_{prediction}$ denotes the vancomycin CL of the individual obtained from the prediction, $CL_{true}$ denotes the actual vancomycin CL of the individua).

Residual plots for each patient regarding actual CL and predicted CL, scatter plots of CL predicted by the PPK method and machine learning combined with the PPK method, and scatter plots with predicted CL as the *x*-axis and actual CL as the *y*-axis.

## 3 Results

### 3.1 Simulated patients’ information

Information on the variables for the 1,000 simulated patients in machine learning is shown in Table 1. The values of the numerical variables (age, weight, serum creatinine) of the simulated patients are within the selected PPK model variable intervals. In the simulated data, the ratio of male to female patients is 1:1.

**TABLE 1:** Information on simulated patients.

Information	Median (range)
Age (years)	54.8 (20.0–90.0)
Weight (kg)	62.5 (40.0–85.0)
serum creatinine (μmol/L)	274.5 (50.0–500.0)

### 3.2 Performance metrics for machine learning models

The results of the performance metrics derived from the four selected machine learning models are shown in Table 2. It should be noted that the MSE, RMSE, and MAE here cannot be used for the final evaluation because the vancomycin clearance rates of the 1,000 patients included in the machine learning are only the reference CL and do not have a direct relationship with the actual CL.

**TABLE 2:** Performance metrics for machine learning.

Model	MAE of test set	*R* ^2^ of trian test	MSE of train test	RMSE of train test
GBDT	2.3204	0.8653	0.7913	0.8896
XGBoost	2.4149	0.9123	0.5151	0.7177
DTR	2.4811	0.8582	0.8325	0.9124
ETR	2.0784	0.8254	1.0253	1.0126

GBDT, Gradient Boosting Decision Tree, XGBoost, eXtreme Gradient Boosting; DTR, Decision Tree Regression; ETR, Extra Tree Regression; MSE, Mean Square Error; MAE, Mean Absolute Error; RMSE, Root Mean Square Error.

As can be seen from Table 2, among the four machine learning models, the model fitted by XGBoost has the highest R-squared of 0.9123 and the lowest MSE and RMSE of 0.5151 and 0.7177, respectively, which indicate that the model fitted by XGBoost is the most effective. Considering that XGBoost do not perform badly in the test set and that the test set here is not used for final evaluation, the XGBoost model is selected as the final machine learning model.

### 3.3 Performance metrics for machine learning models

**FIGURE 2:**
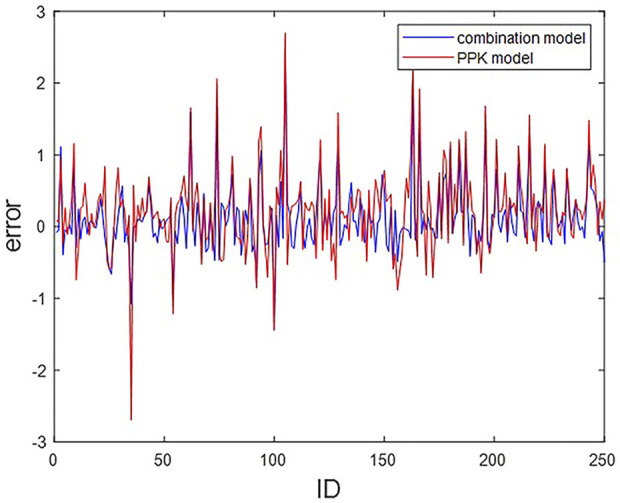
Residual plots of CL predictions from PPK and combined models. The horizontal coordinate indicates ID of the 250 patients; The vertical coordinates indicate the residuals of the two models.

We compare the machine learning combined with PPK method CL prediction model in this paper with the original PPK model. The residual plots of the two prediction methods regarding the actual CL and the predicted CL are shown in Figure 2.

Statistically, among the 250 subjects, a total of 204 subjects predicted the CL value through the combination model, which is closer to the actual CL value than the CL value predicted by the PPK model, accounting for 81.6%. That is, more than four-fifths of the subjects are improved by the combined model.

**FIGURE 3:**
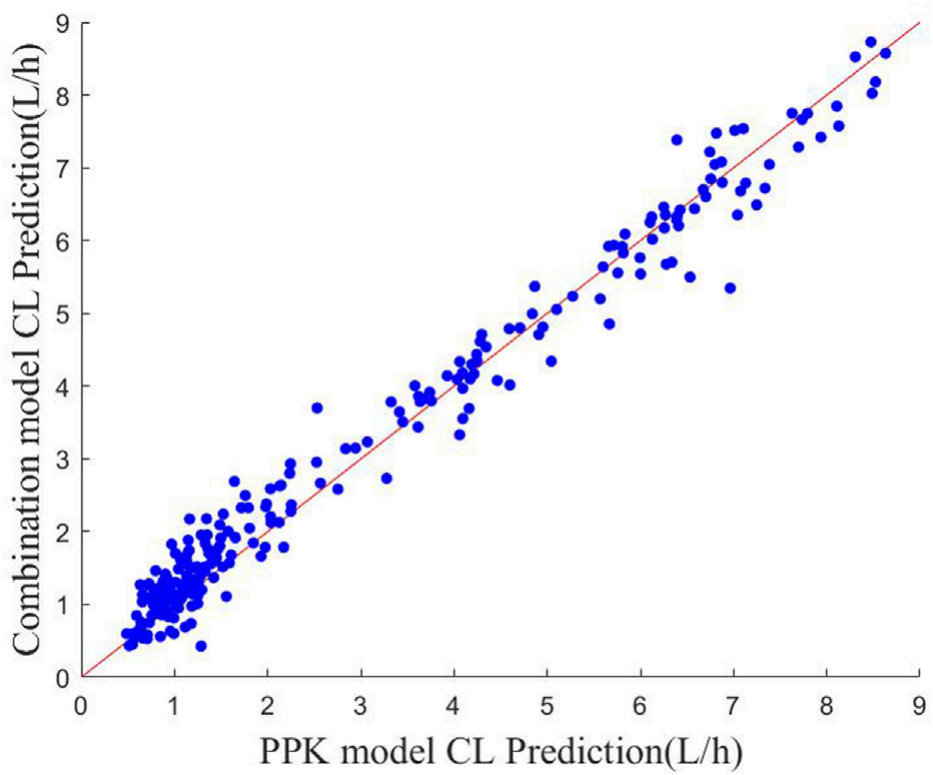
Scatter plot of CL predictions for the two models The red line indicates the y = *x* polyline.

The scatter plots of the predicted CL values for the two methods are shown in Figure 3. It can be found that most of the scatter points are approximately distributed around the y = *x* auxiliary line, which indicates that the CL values predicted by the PPK method and machine learning combined with the PPK method are relatively close to each other. It reflects that the fitting effect of the prediction model of machine learning combined with the PPK method is better from the side.

**FIGURE 4:**
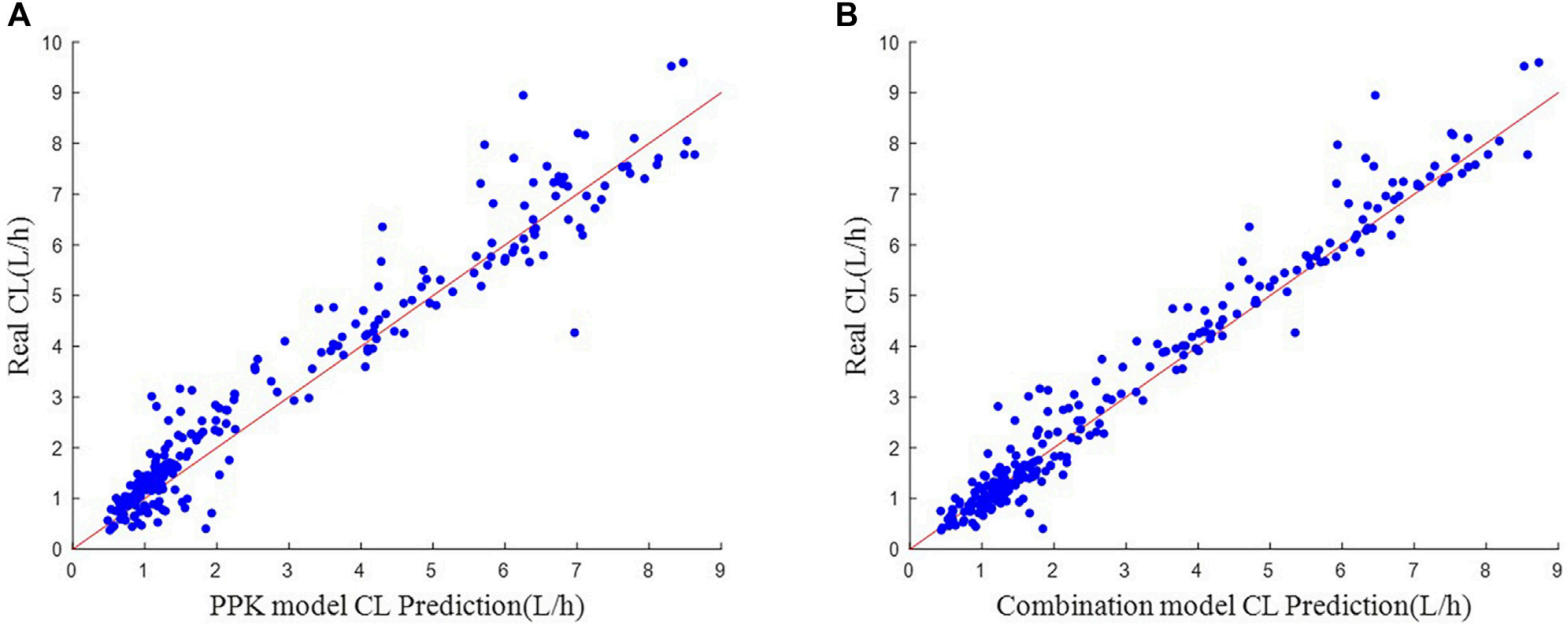
Scatter plot of actual-predicted CL values. **(A)** Real CL vs. PPK model CL Prediction. **(B)** Real CL vs. Combination model CL Prediction.

The scatter plot of actual-predicted CL values for vancomycin in subjects is shown in Figure 4. Comparing Figures 4A, B, the scatters of both plots hover around the neighborhood of the y = *x* auxiliary line. But the scatters in (B) are more uniformly distributed near the y = *x* auxiliary line, and the deviation is not as large as in (A). This shows that the machine learning combined with PPK method prediction model is more stable in performance than the PPK prediction model.

The mean and range of intervals of absolute and relative errors of the two prediction models are shown in Table 3. It can be found that the upper quartile of the absolute or relative error of the prediction model of the machine learning combined with PPK method is even smaller than the mean of the absolute or relative error of the prediction model of the PPK method among 250 subjects. This shows that the combined model is superior to the PPK model. Both in absolute and relative errors, the combined model is much smaller than the PPK model.

**TABLE 3:** Absolute and relative errors of the two models.

Modal	Absolute error		Relative error	
	Mean	Interquartile range	Mean	Interquartile range
PPK	0.4567	(0.1906,0.5674)	0.2183	(0.0777,0.2774)
ML combined with PPK	0.3171	(0.0999,0.3566)	0.1563	(0.0354,0.1966)

## 4 Discussion

In this study, the original PPK method CL prediction equation is used to obtain the reference CL of adult patients, and then the XGBoost model is selected for machine learning to finally obtain the machine learning combined PPK method CL prediction model in this paper. Compared with the original PPK model, the combined model performs better in prediction effect.

In the simulated data, the values of each variable are within the variable range of the PPK model, and it can be seen in Figure 4A that the CL predicted by the PPK model is relatively close to the real CL. In Table 3, the PPK model has a mean absolute error of 0.4567 and a relative error of 0.2183, which is already a good result. In terms of the selection of machine learning models, we choose XGBoost as the final model. By introducing XGBoost based on PPK, the combined model formed is not much different from the CL calculated by the PPK model (Figure 3). However, it is pleasantly surprised that the combined model produced better results. As can be seen in Figure 2, out of 250 patients, the combined model has most lower errors than the PPK model. A total of 204 patients were statistically improved in CL prediction. Comparing Figures 4A, B, it can also be seen that the combined model is closer to the CL in the simulated data. In Table 3, the combined model is better than the PPK model in terms of both absolute and relative errors.

Since the introduction of population pharmacokinetics in the 1970s (Sheiner et al., 1977), PPK has been widely used to guide new drug development. Individual pharmacokinetics can be characterized, and the pharmacokinetic behavior of many individuals can be expressed by quantifying covariates with known sources of variability (Koch et al., 2020). Despite the continuous developmental advances in PPK, predicting parameters using PPK models is still challenging in terms of accuracy. Due to various limitations, only sparse samples can be collected from individuals, which makes the accuracy of pharmacokinetic parameter estimation notoriously compromised.

Machine learning can handle many predictors and allows the use of new types of data (Obermeyer and Emanuel, 2016). It is a data-driven approach and is not based on the results of programming. In recent years, machine learning methods have become increasingly popular in various fields. In fuel cell research, machine learning algorithms can be successfully used for performance prediction, lifetime prediction, and fault diagnosis of fuel cells, with good accuracy in solving nonlinear problems (Su et al., 2023); machine learning has become one of the most promising research methods in novel material screening and material performance prediction (Huang et al., 2023); and in healthcare, machine learning has been used to examine health-related data, and medical professionals can enhance diagnosis and treatment through machine learning applications (An et al., 2023).

Currently, machine learning performs well in the prediction of pharmacokinetic parameters. For example, it can be used to predict drug the area under the curve (AUC) (Bououda et al., 2022; Destere et al., 2023). Recently, it is found that machine learning can individualize vancomycin dose in neonates (Tang et al., 2023). PPK can take advantage of the basic knowledge of physiology and pharmacology development, while machine learning models can improve the accuracy of prediction, and combining the two can produce very impressive results. The use of machine learning combined with population pharmacokinetic methods has been reported to improve the estimation of individual iohexol clearance (Destere et al., 2022). Recently, a study developed a prediction model for Tacrolimus CL in children with Nephrotic Syndrome using machine learning combined with PPK method, which provides a powerful tool for individualized treatment of TAC in pediatric Nephrotic Syndrome patients (Huang et al., 2022).

Machine learning approaches have been widely adopted within the early stages of the drug discovery process, particularly within the context of small-molecule drug candidates. Despite this, the use of ML is still limited in the pharmacokinetic/pharmacodynamic (PK/PD) application space (Pillai et al., 2022). PK/PD analysis using Pharmacometrics provides mechanistic insight into biological processes but is time- and labor-intensive. In contrast, ML models are much quicker trained, but offer less mechanistic insights. The opportunity of using ML predictions of drug PK as input for a PK/PD model can strongly accelerate analysis efforts (Keutzer et al., 2022). The future application of machine learning in predicting drug PK/PD is very promising, and it is expected to bring more efficient solutions to the field of drug development.

Based on the above developments and challenges, this paper explores whether the prediction of vancomycin CL can be improved by machine learning combined with the PPK method in adult patients. This method has not been similarly studied in adult patients. The results of the study show that the machine learning combined with PPK method has a smaller error than the PPK method alone (Figure 2), and the machine learning combined with PPK method performed better in both absolute and relative errors (Table 3), reflecting that the machine learning combined with PPK method can improve the prediction of vancomycin CL in adult patients in China.

This study does have some limitations as well. First, since the study data is simulated, this may lead to some deviations from real patients. Second, we choose four machine learning models for comparative analysis based on a rule of thumb and literature review, and ultimately choose the XGBoost model, but it is possible that better machine learning models exist that can replace the XGBoost model. In addition, because four covariates are selected in population pharmacokinetics, machine learning is only analyzed for these four covariates to make predictions, and it is possible that there are other covariates that can be subjected to machine learning but have been neglected. Future studies should combine population pharmacokinetics and machine learning analysis methods to find better machine learning models to assess the impact of covariates on predicting individual clearance.

## 5 Conclusion

In summary, this paper combines machine learning with population pharmacokinetics, and ultimately find that the application of machine learning combined with population pharmacokinetics can improve the prediction of vancomycin clearance in Chinese adult patients. It provides a reference for clinical pharmacists or clinicians to optimize the initial dosage given to ensure the effectiveness and safety of drug therapy for each patient.

## Data Availability

The raw data supporting the conclusion of this article will be made available by the authors, without undue reservation.
